# Small Animal Veterinarians' Communication With Dog Owners From a Motivational Interviewing Perspective

**DOI:** 10.3389/fvets.2021.772589

**Published:** 2021-11-25

**Authors:** Karolina Brunius Enlund, Ebba Jennolf, Ann Pettersson

**Affiliations:** Department of Clinical Sciences, Swedish University of Agricultural Sciences, Uppsala, Sweden

**Keywords:** veterinary communication, veterinary dentistry, dental health, dental home care, dog, MI

## Abstract

Veterinary communication skills are fundamentally important in animal practice. Despite client-centered communication being recommended as the optimal medical communication style, a paternalistic approach is still common in veterinary medical encounters with pet owners. Motivational interviewing (MI) is a client-centered, evidence-based counseling method aiming to strengthen a person's motivation and commitment to behavior changes. In this exploratory study, the aim was to investigate Swedish small animal veterinarians' use of client centered communication with dog owners regarding dental home care in dogs. This was achieved by analyzing the use of MI-techniques among veterinarians without previous training or knowledge of the method. Individual telephone calls, reflecting a veterinary clinical scenario, between small animal veterinarians (*n* = 8) and a trained professional actor playing a dog owner were recorded and coded according to an MI coding protocol (MITI 4.2.1). In the present study, the degree of spontaneously used MI was low. From an MI-communication perspective, with a simulated dog owner, the veterinarians predominantly relied on asking questions, giving information, and persuasive talk. The veterinarians dominated the conversations and made minimal attempts to involve the dog owner resulting in a power imbalance between veterinarian and client. As the degree of spontaneously used MI was found to be low, MI-training may be required in order to apply the method in professional counseling. The veterinarians' communication pattern suggested a paternalistic communication style, when attempting to motivate a client to brush his or her dog's teeth. We suggest that Motivational Interviewing (MI) has a potential to improve veterinary communication and adherence to medical recommendations if introduced and implemented in veterinary practice.

## Introduction

Effective communication is an important skill in veterinary practice. Veterinarians, in their professional role, not only need to gather and provide information effectively, but also to motivate animal owners to implement behavior changes, aiming toward improving the health of their animals, e.g., regarding diet, medication, or home care (1).

Traditionally, the most common communication approach used in a clinical setting is paternalism, in which the veterinarian sets the agenda for the appointment, assumes that the client's values are the same as the veterinarians, and takes on the role of a guardian (2–5). In such a relationship, the veterinarian performs most of the talking, while the client has a more passive role, the focus being medical condition, diagnosis, treatment, and prognosis. This persuasive approach has been proven to be ineffective and to increase resistance to change in ambivalent clients (6).

Instead, client-centered communication has been suggested as the optimal medical communication style, where focus lies on partnership and shared decision-making, with the clinician acting as an advisor or counselor (7). Client-centered communication has been the focus in several communication models, e.g., The Four Habits model, the Calgary Cambridge guide, and Motivational Interviewing (MI) (8–10). However, data suggest that a biomedical or paternalistic approach is still the most common way of communicating in veterinary practice, and the practice of client-centered communication in veterinary care remains inadequate (4, 11, 12).

In human health care, the application of the evidence-based counseling method called MI is increasing (13). In contrast to paternalism, MI is a collaboration centered counseling style aiming to strengthen a person's inner motivation and commitment to change (10). Within MI, the clinician acts like more of a guide than an advisor, helping the client to resolve how he or she should act instead of telling the client what to do. MI is distinguished from other counseling styles especially through *Cultivating Change Talk*, i.e., to promote language a client uses that favors change, and *Softening Sustain Talk*, i.e., to decrease a client's language in favor of the status quo, using the core skills Open Questions, Affirming, Reflecting and Summarizing (OARS) (10). Studies have shown that this style of communication may be an effective way to promote behavior changes in clients (13, 14).

MI originates from experiences of alcoholism treatment (10). Today, MI has been used in many different contexts requiring lifestyle changes, such as treatment of substance misuse, weight loss, dental care, and medication adherence (13, 15–17). However, studies regarding MI in veterinary care are few (12, 18–21), although promising results have been presented for communication between cattle farmers and veterinarians, where MI has been shown to increase adherence to veterinary recommendations for herd health management (19). In addition, training in MI improved veterinarians' communication skills and was considered by veterinarians as highly relevant and useful for their profession (18).

The extent to which MI is used during a counseling session is measured in a standardized protocol called Motivational Interviewing Treatment Integrity 4.2.1 (MITI 4.2.1) (22). The MITI protocol consists of two main parts: the global scores and the behavior count (Supplementary Material). In this work, we consistently refer to MITI protocol components in italics. The global scoring is based on coder assessment of technical components (*Cultivating Change Talk, Softening Sustain Talk*) and relationship components (*Partnership, Empathy*). These components are scored from one (least) to five (most) with three as default reference value. For behavior counts, the coder counts the instances of 10 specific behaviors during the conversation. Each instance of the particular behavior is counted without any further judgement. The behaviors included are *Giving Information, Persuade, Persuade with Permission, Question, Reflection Simple, Reflection Complex, Affirm, Seeking Collaboration, Emphasizing Autonomy, Confrontation*. Of these behaviors, *Persuasion* and *Confrontation* are MI-non-adherent behaviors, while the remaining behaviors are more or less MI-adherent. A random sample of the conversation lasting for 20 min is recommended for coding (22).

Poor dental health is one of the most common health issues in small animal medicine. Several studies report that periodontal disease is the most common disease in dogs over 3 years of age, with a prevalence ranging between 80 and 89% (23–26). The condition is particularly common in toy and small dog breeds (27–29), and the incidence increases with age (26, 27). Due to inadequate owner awareness and subtle outwardly detectable signs, periodontal disease is a severely under-diagnosed and therefore undertreated condition in dogs, despite the high prevalence (30). Gold standard for the prevention of periodontal disease is daily tooth brushing, however, tooth brushing in dogs requires good owner adherence (30). Although few studies have been performed, daily tooth brushing in dogs is likely to be rare (31).

This study aimed to investigate the extent to which, and in what way, Swedish small animal veterinarians, without previous training or knowledge of the method, spontaneously use MI in their communication with dog owners regarding dental home care in dogs.

## Materials and Methods

Telephone calls, reflecting a veterinary clinical scenario, between small animal veterinarians and a professional actor playing a dog owner were recorded during September and October 2020. The audio files were sent to MIC Lab AB, Stockholm (https://miclab.se), for coding according to MITI 4.2.1.

### Motivational Interviewing Treatment Integrity 4.2.1

All conversations were coded according to the manual for MITI 4.2.1 at MIC Lab AB, a Swedish center of expertise for quality assurance, coding, and education in MI. Reliability of the coders is verified through regular controls. To minimize inter-rater bias, all conversations were coded by the same coder. In addition to the coding, written feedback from the coder was received from the coder for each conversation, with suggestions for improvements from an MI-perspective.

### Participating Veterinarians

In total, eight veterinarians were recruited to the study through convenience sampling, seven women and one man with an average age of 41 years, ranging between 31 and 52 years. All participants graduated as veterinarians during the period 2000-2018 and had been working as clinical small animal veterinarians for 10 years on average, ranging between 3 and 20 years. Three of the veterinarians had not participated in any further education in dentistry, while the remaining participants had completed at least one course within veterinary dentistry after completing their veterinary education. None of the veterinarians had participated in any form of communication training.

### The Conversations

Each veterinarian participated in one telephone conversation with a standardized client played by the same actor (male) employed by MIC Lab AB. The actor was trained for this purpose and had experience in role-play scenarios using MI. The actor did not use a script but was provided with a client profile with background information about the dog owner and the dog. The client profile was developed based on literature studies and clinical experience, and in collaboration with Lars Forsberg at MIC Lab AB. In short, the scenario was: A 6-year-old female cocker spaniel visited the veterinary clinic for professional dental cleaning and was diagnosed with periodontitis. Several teeth had been deemed unsalvageable because of severe periodontitis and extracted. To stop disease progression, implementation of daily tooth brushing is now necessary. The dog owner's willingness to start brushing the dog's teeth was scored nine on a ten-point scale, and his confidence in his ability to succeed was scored two on a ten-point scale.

General information about the nature of the study, a communication study with an actor playing a dog owner, and background information about the scenario, was sent in advance to participating veterinarians *via* e-mail.

Before each conversation, the actor telephoned the veterinarian to confirm that the veterinarians had received information about the study, that the call would be recorded, and that they consented to participate in the study. The actor then telephoned again as the fictional dog owner and recorded the call using a digital voice recorder. The calls were directed by the actor to be ~20 min long to allow recommended coding time. After each conversation, the actor uploaded the audio file to the website of MIC Lab AB, from where the MITI-coder could access them.

### Data Management

For each recorded call, a coding protocol was obtained from MIC Lab AB. Based on the obtained coding protocols mean values ± standard deviation for the global scores and behavior counts were calculated. The coder's written comments were compiled by the author E.J. and presented in text.

## Results

### Global Scores and Behavior Counts, MITI 4.2.1

Descriptive statistics (mean ± standard deviation) of the global scores and behavior counts are presented in Table 1. The global scores were ≤ 3 (reference default value) in all coding protocols. *Cultivating Change Talk* was scored lowest possible (score one) in all conversations. The *Softening Sustain Talk* scores were either two (6/8) or three (2/8). The *Partnership* scores were also either two (6/8) or three (2/8). *Empathy* was scored two in all (8/8) coding protocols.

**Table 1:** Descriptive statistics (mean ± standard deviation) of 14 variables (global scores and behavior counts) describing MI-skills according to MITI 4.2.1.

	**Variable**	**Mean (SD)**
Global scores (1–5)^*^	Cultivating change talk	1 (±0)
	Softening sustain talk	2.25 (±0.46)
	Partnership	2.25 (±0.49)
	Empathy	2 (±0)
Behavior counts	Giving information	8.25 (±2.12)
	Persuade	6.50 (±1.69)
	Persuade with permission	2.25 (±1.16)
	Questions	3.13 (±1.25)
	Simple reflection	1.25 (±0.71)
	Complex reflection	0.88 (±0.64)
	Affirm	0.75 (±0.71)
	Seeking collaboration	0.63 (±0.74)
	Emphasizing autonomy	0 (±0)
	Confront	0 (±0)

* *Three is considered the default global score*.

The veterinarians' consultation approaches were predominantly characterized by *Giving information, Persuasion*, and *Questions* (Table 1). On a few occasions, the veterinarians used *Persuasion with Permission*. All veterinarians made at least one *Simple* or *Complex reflection* in response to client statements. The participants made few attempts to *Affirm* the client, and few were *Seeking Collaboration*. None of the veterinarians were *Emphasizing Autonomy* during the calls. The behavior *Confront* was not shown (Table 1). Overall, the results were very similar for all participating veterinarians.

### Written Comments

In the written comments, the coder noted both the clinical strengths of the veterinarians and potential areas for improvement in terms of MI, by commenting how behaviors in the behavior count affected the global scores.

The coder identified that most of the veterinarians were asking *Questions* trying to understand the client's situation and thoughts, positively affecting the *Empathy* score. Actual examples include:

- “What do you think about that, may that work?”
- “Have you brushed her teeth…?
- “Is it hard and troublesome to do so…?”
- “How is it going with the tooth brushing?”
- “Have you been able to start brushing her teeth?”
- “Does she think it is scary?”

The coder noted that *Reflections*, such as “Just as you said, daily tooth brushing…” also positively affected the *Empathy* score. Another clinical strength was that the veterinarian in a few situations *Affirmed* the dog owner by acknowledging the client's achievements, which positively affected the *Partnership* score.

The coder also suggested areas for improvement from an MI-perspective. Regarding *Cultivating Change Talk*, the score would have been higher if the veterinarians would have asked the client about the reasons why the change was needed. This was noted in 8 of 8 conversations.

The *Partnership* scores were adversely affected by the veterinarians (8/8) giving advice without the client's permission (*Persuade*). Additionally, all the veterinarians (8/8) dominated the conversations having most of the speaking time. The *Partnership* scores would have been positively affected if the veterinarians had involved the client more in the conversation and *Sought Collaboration* to a greater extent. In more than half of the protocols (6/8), the coder also mentioned that the veterinarian could have *Emphasized Autonomy* to strengthen the *Partnership* with the client.

Another comment noted by the coder regarded the behavior *Affirm*. To achieve a higher score in *Partnership*, the veterinarians could have affirmed the client when expressing ambitions to make a change and acknowledged the client's efforts and achievements. To achieve a higher score in *Empathy*, the veterinarians could have made more *Simple* or *Complex reflections* on the client's utterances.

## Discussion

### Characterization of Communication Styles

To the authors' knowledge, this is the very first time that the degree of spontaneous use of MI in small animal veterinarians without prior training has been investigated.

In the present study, the global scores were consistently low (≤ 3). *Cultivating Change Talk* was scored the lowest possible in all conversations, which implies that the veterinarians showed no explicit attention to, or preference for, the client's language in favor of changing according to the MITI 4.2.1 manual (32). Regarding *Softening Sustain Talk*, the majority (6/8) of the veterinarians unfortunately mainly chose to explore, focus on, or respond to the client's language in favor of the status quo. However, some (2/8) of the participating veterinarians made attempts to shift focus away from the sustain talk. The scores would likely increase if the veterinarians became more aware of sustain talk and how to respond in favor of change.

*Partnership* and *Empathy* have been identified as critical elements of a relationship-centered consultation approach (10). In addition, partnership and shared decision making (i.e., healthcare professional together with client reaching a decision about care) in veterinary care is favored by both veterinarians and pet owners according to recent research (33, 34). However, the veterinarians in the present study predominantly superficially responded to opportunities to collaborate, resulting in low *Partnership* scores. Although some veterinarians (2/8) incorporated the client's contributions to some extent. Moreover, *Empathy* was equally scored two in all coding protocols meaning that the veterinarians only made sporadic efforts to explore the client's perspective. Since low advisor empathy has been identified as damaging to the advisor relationship and associated with poorer patient outcomes (35), the authors suggest empathy as an area for improvement.

Based on the behavior counts, the conversations were dominated by the veterinarians relying predominantly on *Giving information, Persuasion*, and *Questions* in their communication with the dog owner. According to the MITI 4.2.1 manual, this implies that the veterinarians focused primarily on providing information and educating the client, making overt attempts to change the client's attitude, opinions, or behavior, and eliciting information by asking questions (32). Occasionally, the veterinarians used *Persuasion with Permission*, which means that they included an emphasis on collaboration or autonomy support while persuading. This behavior is considered an MI-adherent behavior, in contrast to *Persuasion*. These results are in accordance with the findings of previous studies (4, 11, 12, 19) and suggest that a paternalistic communication style was adopted by the participating veterinarians taking on the role of an expert paid to provide a service of advice and solutions. In terms of health related problem appointments, a paternalistic approach was used by the veterinarian in 85% of the cases, and by 54% of wellness appointments (4). The results presented in the present study may indicate that the paternalistic communication style is frequently used also among Swedish small animal veterinarians, even though this persuasive approach has been proven to be ineffective and also increases resistance to change in ambivalent clients (6). Persuasion is more likely to elicit reactions against, rather than in favor of, change (36). This phenomenon is known as psychological reactance and has been a frequent subject for research since the reactance theory was first described by Brehm (37). In addition, studies in medical communication have shown a positive association between the use of relationship-centered care and aspects of clinical outcomes, such as patient satisfaction, patient health outcomes, physician satisfaction, and reduction of malpractice complaints (4).

The veterinarians in the study relied heavily on *Questions*, but only one of the questions that the coder highlighted was an open question. The remaining were closed questions with limited answer options such as “yes,” “no,” “maybe,” or “do not know.” In contrast to closed questions, open questions stimulate reflection and exploration in favor of change (10). By reformulating the questions into open questions, there is a potential to evoke change talk, strengthen partnerships, and thus, promote behavior change.

In general, the core skills of MI are not considered to be regularly used in professional conversations (10), which was also true in the present study. The veterinarians practiced reflective listening to a very small extent. However, all veterinarians made at least one *Simple* or *Complex reflection* in response to client statements. Likewise, the veterinarians made few attempts to *Affirm* the client by accentuating something genuinely positive about the client's strengths, efforts, intentions, or worth (32). These results suggest that there is potential for improvement regarding several behaviors. Encouragingly, all these behaviors can be improved by communication training.

Furthermore, few of the veterinarians were *Seeking Collaboration*, which implies making attempts to share power or acknowledge the expertise of the client (32). Without seeking collaboration, the veterinarian controls the conversation supposing and communicating to have the best solutions to the client's problems, resulting in unequal power distribution between veterinarian and client. Such an approach has been shown to be ineffective and to increase resistance to change in ambivalent clients (6). Contrarily, a more client-centered communication style may be beneficial to achieve behavior change (14). According to research guided by the self-determination theory (SDT), autonomy is one of three innate psychological needs, which when satisfied, enhances self-motivation, and when thwarted leads to diminished motivation (38). When comparing people whose motivation is authentic with those who are simply externally controlled for action, the former, relative to the latter, typically have more interest, excitement, and confidence. This in turn leads to enhanced performance, persistence, and creativity. However, none of the veterinarians in the present study were *Emphasizing Autonomy* by making utterances that highlight the client's freedom of choice and right to make his own decisions about his dog. This may lead to decreased client motivation and shows that there is room for improvement in veterinary communication.

Similar to the present study, a predominance of MI-nonadherent behaviors has been reported in studies of food safety by health and environmental inspectors (39) as well as cattle veterinarians (12, 19). Thus, MI-training is required to be able to apply the method in professional counseling (10). Studies have shown that considerable time is required to learn MI (10, 39). To read or hear about the method is seldom enough; to develop MI-skills, practical training, including feedback, is needed. However, communication training seems to be in demand among veterinarians. In a survey among veterinary practitioners in the United Kingdom and the United States in 2012/2013, 40% answered that they would be interested in further veterinary communication skills training, with the preferred methods being simulated consultations and online training (40). In a recently published study, Svensson et al. (18) evaluated a 6-month training program in MI for veterinarians involved in veterinary herd health management. After completing the training program, all participating veterinarians had significantly improved their MI-skills, regarding at least one parameter. Veterinarians with higher MI-skills have in turn been associated with increased expression of so-called change talk from the client (21). Moreover, the participating veterinarians perceived their new skills and knowledge of MI as highly relevant in their work (18). Likely, MI-training of small animal veterinarians would have similar effects.

Encouragingly, no veterinarians showed *Confronting* behaviors such as disagreeing, arguing, shaming, blaming, criticizing, moralizing, or warning the client which are considered MI-nonadherent behaviors (32), and could be very counterproductive in terms of behavior change.

### Dental Home Care Recommendations

The predominance of *Giving information, Persuasion*, and *Questions*, characteristic of paternalism, in the consultation approach among veterinarians, might create psychological resistance, and may be contributing to the low uptake of dental home care recommendations reported. One previous study showed that adherence to dental homecare instruction in dogs that had undergone periodontal surgery was low, with only 24% of dog owners still brushing daily after 6–20 months post-op (41). In addition, we have in a recent survey of preventative dental home care in dogs in Sweden shown that <4% of Swedish dog owners brushed their dog's teeth daily, and that adherence to veterinary recommendation about tooth brushing was low. Nevertheless, the majority of the dog owners answered that they might consider brushing, indicating a great potential motivation to perform dental home care (31). In human dentistry, MI is recommended in the national guidelines as a method for effective dental care communication (17). Similarly, the implementation of MI in veterinary practice may be a tool to improve adherence regarding tooth brushing in dogs.

The field of application for MI in veterinary practice is extensive. MI may be applied in every situation where a change in the behavior of the animal owner is desirable, such as weight loss, medication, and rehabilitation, as well as dental home care. We suggest that MI may be helpful in increasing adherence to veterinary recommendations in all these fields.

### Methodological Considerations

In the present study, role-play scenarios with a professional actor were used instead of real clinical situations to standardize the degree of difficulty and conditions for practicing MI as far as possible. By using role-play scenarios with the same actor in all conversations, the variability was minimized, and the reliability increased. In turn, the validity of the obtained results increased. Moreover, all calls were coded by the same coder at MIC Lab AB to avoid the risk of inter-rater bias. The intra-rater reliability was considered high as the coders at MIC Lab AB undergo continuous training and regular controls.

However, the MITI scores may not only vary depending on the external conditions but also on the veterinarian's daily form. This implies that the estimate of the veterinarians' true MI-skills would have been improved if multiple calls with each veterinarian had been coded. Furthermore, the number of calls in this study was very limited, thus further studies with a larger scope are required to be able to characterize the communications styles of Swedish small animal veterinarians in general. In addition, the sample of veterinarians was not chosen at random which implies potential bias due to over- or under-representation of subgroups in the sample compared to the target population. For these reasons, the sample of the present study may not be representative of the population of Swedish small animal veterinarians, which should be considered when interpreting the results.

The dog owner profile was created in collaboration with the experienced MI-researcher Lars Forsberg at MIC Lab AB, based on literature review and clinical experience of a typical dog and owner. There is likely a variation in the veterinarian's communication depending on the pet owner, hence the results may have been different if the dog owner profile, or the actor, would have been another. It should, however, be noted that the conversations with the actor were experienced by all participating veterinarians as completely natural and resembling a real clinical situation.

## Conclusion

In the present study, the degree of spontaneously used MI is low, implicating that MI-training is required to apply the method in professional counseling. From an MI-communication perspective, with a simulated dog owner, the veterinarians predominantly relied on asking questions, giving information, and persuasive talk, suggesting a paternalistic communication style, when attempting to motivate a client. The veterinarians dominated the conversations and made minimal attempts to involve the dog owner resulting in a power imbalance between veterinarian and client. We suggest that veterinary communication and thereby client adherence to medical recommendations may improve if a client-centered communication style such as MI is introduced and implemented in veterinary practice.

## Data Availability Statement

The original contributions presented in the study are included in the article, further inquiries can be directed to the corresponding author/s.

## Ethics Statement

Ethical review and approval was not required for the study on human participants in accordance with the local legislation and institutional requirements. The patients/participants provided their written informed consent to participate in this study.

## Author Contributions

KE and EJ: conceptualization and original drafting of the manuscript. EJ: data collection and analysis. AP: review and editing. All authors approved the submitted version.

## Funding

This work was supported by the Swedish University of Agricultural Sciences (SLU) and the Swedish Association for the Protection of Animals (Svenska Djurskyddsföreningen).

## Conflict of Interest

The authors declare that the research was conducted in the absence of any commercial or financial relationships that could be construed as a potential conflict of interest.

## Publisher's Note

All claims expressed in this article are solely those of the authors and do not necessarily represent those of their affiliated organizations, or those of the publisher, the editors and the reviewers. Any product that may be evaluated in this article, or claim that may be made by its manufacturer, is not guaranteed or endorsed by the publisher.
