# Supplementary material for: Small Animal Veterinarians' Communication With Dog Owners From a Motivational Interviewing Perspective
Source: Front Vet Sci. 2021 Nov 25;8:772589. doi: 10.3389/fvets.2021.772589 (PMC8655983; doi:10.3389/fvets.2021.772589)
Supplement: Supplementary file 1 [file Table_1.DOCX]

*Small animal veterinarians’ communication with dog owners from a Motivational Interviewing (MI) perspective*

Supplementary material

Schematic table of Motivational Interviewing Treatment Integrity (MITI) 4.2.1 protocol (1).

| Global scores (score 1-5) |  |  |
| --- | --- | --- |
|  | *Technical components* | Cultivating Change Talk |
|  |  | Softening Sustain Talk |
|  | *Relationship components* | Partnership |
|  |  | Empathy |
| Behavior counts |  |  |
|  |  | Giving information |
|  |  | Persuade |
|  |  | Persuade with permission |
|  |  | Question |
|  |  | Reflection Simple |
|  |  | Reflection Complex |
|  |  | Affirm |
|  |  | Seeking collaboration |
|  |  | Emphasizing Autonomy |
|  |  | Confrontation |

Reference:

1. Moyers TB, Rowell LN, Manuel JK, Ernst D, Houck JM. The motivational interviewing treatment integrity code (MITI 4): rationale, preliminary reliability and validity. Journal of substance abuse treatment. 2016;65:36-42.
